# Genome-Edited, TH-expressing Neuroblastoma Cells as a Disease Model for Dopamine-Related Disorders: A Proof-of-Concept Study on DJ-1-deficient Parkinsonism

**DOI:** 10.3389/fncel.2017.00426

**Published:** 2018-01-08

**Authors:** Jannik Prasuhn, Christoph U. Mårtensson, Victor Krajka, Christine Klein, Aleksandar Rakovic

**Affiliations:** Institute of Neurogenetics, University of Lübeck, Lübeck, Germany

**Keywords:** genome editing, dopamine metabolism, TH, Parkinson’s disease, DJ-1

## Abstract

Impairment of the dopaminergic (DA) system is a common cause of several movement disorders including Parkinson’s disease (PD), however, little is known about the underlying disease mechanisms. The recent development of stem-cell-based protocols for the generation of DA neurons partially solved this issue, however, this technology is costly and time-consuming. Commonly used cell lines, i.e., neuroblastoma (SHSY5Y) and PC12 cells are still widely used to investigate PD and significantly contributed to our understanding of mechanisms involved in development of the disease. However, they either do not express DA at all or require additional, only partially efficient differentiations in order to produce DA. Here we generated and characterized transgenic SH-SY5Y cells, ectopically expressing tyrosine hydroxylase (SH^TH+^), that can be used as a homogenous, DA-producing model to study alterations in DA metabolism and oxidative stress. We demonstrated that SH^TH+^ produce high levels of DA, 3,4-dihydroxyphenylacetic acid (DOPAC), and homovanillic acid (HVA) making this model suitable to investigate not only alterations in DA synthesis but also its turnover. We also provide evidence for the presence of other enzymes involved in DA synthesis and its turnover in these cells. Finally, we showed that these cells can easily be genetically modified using CRISPR/Cas9 technology in order to study genetically defined forms of movement disorders using DJ1-linked PD as a model.

## Introduction

Parkinson’s disease (PD) is the second most common neurodegenerative disorder, clinically characterized by the combination of bradykinesia, tremor, rigidity and postural instability caused by loss of dopaminergic neurons in substantia nigra (Postuma et al., 2015). However, the specific vulnerability of dopaminergic neurons as the underlying disease mechanism has not yet been sufficiently explained. Induced pluripotent stem cells (iPSCs) are the current gold standard for *in vitro* studies of neurological disorders, however, technically complex, labor-intensive, and expensive. Additionally, the turn-around time from pre-experimental setups to subsequent results is substantial compared to experiments performed on primary cells or established cell lines. Although SH-SY5Y cells are a well-established and still commonly used *in vitro* model for neurological disorders, it remains mostly elusive which kind of neurotransmitters they produce precisely. Prior studies have shown that they are able to synthesize and release small amounts of dopamine (DA) however, this is still under debate. Furthermore, they express DA receptors and transporters which are characteristic features of dopaminergic cells in the central nervous system and crucial for DA homeostasis in neurons (Xicoy et al., 2017). More recently, it has been shown that dopamine oxidation represents an important link between mitochondrial and lysosomal dysfunction in PD pathogenesis (Burbulla et al., 2017). Since DA metabolism-induced oxidative stress is a unique feature of DA neurons (Meiser et al., 2013), our goal was to generate and characterize dopaminergic SH-SY5Y cells which would more faithfully recapitulate the underlying mechanisms of PD and to subsequently facilitate the development of new treatment approaches.

Although only 2%–3% of PD cases are caused by monogenetically inherited mutations in PD-associated genes, functional studies of the clinically very similar monogenic forms may serve as a model to gain deeper insights into the underlying pathogenesis of sporadic, idiopathic PD (Lill, 2016). We here present an easy-to-modify dopaminergic SH-SY5Y cellular model and apply it as a proof of concept to the study of DA metabolism in DJ1-linked PD using CRISPR/Cas9 technology. Loss of DJ-1 has been previously linked to altered dopamine turnover and oxidative stress (Raman et al., 2013).

## Materials and Methods

### Cell Culture

Commercially available neuroblastoma (SH-SY5Y) cells (ATCC^®^ CRL-2266™), control human dermal fibroblasts (obtained and established in the Institute of Neurogenetics, Lübeck, Germany), human embryonic kidney (HEK293) cells (ATCC^®^ CRL-1573™), and HeLa cells (ATCC^®^ CCL-2™) were grown at 37°C under a 5% CO_2_ humidified atmosphere in Dulbecco’s Modified Eagle Medium (DMEM, Thermo Fisher Scientific) supplemented with 10% fetal bovine serum (FBS, PAA laboratories) and 1% penicillin/streptomycin (Thermo Fisher Scientific). The number of passages were matched for each experiment.

iPSC-derived dopaminergic neurons were generated using a previously published protocol (Rakovic et al., 2013).

### Generation of Tyrosine Hydroxylase (TH)-expressing SH-SY5Y Cells

To produce tyrosine hydroxylase (TH)-expressing lentiviral particles, a cassette consisting of the open reading frame of the TH gene (NM_199292), the IRES sequence, and the Puromycin resistance gene was cloned into pLenti4/V5-DEST (ThermoFisher) plasmid (pLenti4-TH-IRES-Puromycin). In addition, a cassette containing only the IRES sequence and the Puromycin resistance gene was cloned into pLenti4/V5-DEST plasmid (pLenti4-Empty-IRES-Puromycin). Next, 293FT cells were cotransfected with either pLenti-TH-IRES-Puromycin or pLenti4-Empty-IRES-Puromycin and the ViraPowerTM Packaging Mix (ThermoFisher) to generate a lentiviral stock. SH-SY5Y cells were transduced using either lentiviral particles for 48 h followed by selection using 2 μg/ml Puromycin (ThermoFisher) for a further 48 h.

To produce monoamino oxidase A (MAOA)- or monoamino oxidase B (MAOB)-expressing lentiviral particles, a cassette consisting of the open reading frame of the either the *MAOA* gene (NM_001270458) or the *MAOB* gene (NM_000898), followed by the IRES sequence, and the Blasticidin resistance gene were cloned into pLenti4/V5-DEST (Thermo Fisher) plasmids (pLenti4-MAOA-IRES-Blasticidin and pLenti4-MAOB-IRES-Blastcidin). In addition, a cassette containing only the IRES sequence and the Blasticidin resistance gene was cloned into pLenti4/V5-DEST plasmid (pLenti4-Empty-IRES-Blasticidin). Next, 293FT cells were cotransfected with either pLenti-MAOA-IRES-Blasticidin or pLenti-MAOB-IRES-Blasticidin and the ViraPowerTM Packaging Mix (ThermoFisher) to generate a lentiviral stock. SH-SY5Y cells were transduced using lentiviral particles for 48 h followed by selection using 7 μg/ml Blasticidin S HCl (ThermoFisher) for further 96 h.

Upon selection, cells were grown in DMEM/10%FBS/1%Penicilin/Streptomycin without antibiotics.

### CRISPR/Cas9-mediated Knockout of DJ-1

SH-SY5Y cells were transiently cotransfected with a pLKO1 Puro (modified pLKO.1 vector, Addgene) expressing the gRNA targeting 5′-GTACAGTGTAGCCGTGATG-3′ sequence in exon 3 of the DJ-1 gene and a plasmid expressing spCas9 using a Nucleofection device (Lonza). After 24 h, cells were selected using 2 μg/ml Puromycin (ThermoFisher) for 48 h. Cells were plated on 10 cm petri dishes at a density of 1 cell/cm^2^ and grown until they formed distinct colonies. Part of each colony was used to extract DNA, followed by sequencing of exon 3 of the DJ-1 gene. Successfully edited colonies were further grown to obtain enough cells for the following experiments.

### mRNA Quantification

For mRNA expression studies, total RNA was isolated from SH-SY5Y cells using the RNeasy Mini Kit (Qiagen) and converted to cDNA using the Maxima First Strand cDNA Synthesis Kit for RT-qPCR (Thermo Scientific). Quantitative real-time PCR (qPCR) was performed in duplicates and run on the LightCycler 480 Instrument II (Roche). To compare DJ-1 mRNA levels the primers DJ1 ex4 F 5′-GTGGTGGTTCTACCAGGAGG-3′ and DJ1 ex6 R 5′-TGA GCCAACAGAGCAGTAGG were used. The *DJ1* mRNA expression levels were normalized between samples using the housekeeping gene β-actin as a reference.

### Western Blot Analysis

Proteins were extracted using RIPA buffer (ThermoFisher) and their concentrations were determined using Dc Protein Assay Kit (Biorad). Proteins were separated by SDS PAGE using NuPAGE 4%–12% Bis-Tris gels (ThermoFisher). After electrophoresis, proteins were transferred to a nitrocellulose membrane (Protran) and probed with the antibodies against TH (Millipore, 1:2000), dopa decarboxylase (DDC; Cell Signaling Technology, 1:1000), GTP cyclohydrolase 1 (GCH1; Novus, 1:10,000), DJ-1 (Cell Signaling Technology, 1:100,000), β-actin (Sigma, 1:10,00,000), MAOA (Santa Cruz, 1:2000), MAOB (Santa Cruz, 1:1000). For densitometric analyses, TotalLab software (Nonlinear Dynamics) was used.

### High Performance Liquid Chromatography

Two million cells were harvested, pelleted, washed in 1× phosphate-buffered saline (PBS), and lysed in 200 μL of DA extraction buffer (100 mM perchloric acid, 0.2 mM EDTA) using sonification (2 × 5 s, 50% amplitude, Bandelin). The total amount of DA, 3,4-dihydroxyphenylacetic acid (DOPAC) and homovanillic acid (HVA) was measured by means of high performance liquid chromatography (HPLC) with subsequent electrochemical detection using a C18 column (Eurospher RP 18, particle size 5 pm, column size 250 × 4.0 mm). Additionally, three standard solutions for each, DA, DOPAC, and HVA were prepared and measured subsequently to generate a standard curve. The mobile phase was a degassed solution of 0.15 M sodium acetate buffer, pH 4.0, with 12% methanol, 0.014 g/l EDTA and 0.1 mM sodium 1-octansulfonate and pumped at a flow rate of 1 ml/min. All chromatography experiments were performed at 4°C and all solutions were kept on ice. The separations were achieved under isocratic conditions. The detector cell was operated at +0.8 V.

### Reactive Oxygen Species Measurement

To measure intracellular levels of reactive oxygen species (ROS), CellROX Green Reagent (ThermoFisher) was used. In brief, 100,000 cells were plated into a 96-well microtiter plate 1 day before measurement. On the following day, cells were treated with 5 μM CellROX Green Reagent for 30 min at 37°C. Cells were washed with 1xPBS and fluorescence (Ex/Em 485 nm/520 nm) was measured using a plate reader (BioTek).

### Statistical Analysis

SigmaPlot 11 (Systat Software) was used to perform all necessary statistical analyses. For detailed examination of the observed cell lines, all values are expressed as ratios compared to their respective control. *p*-values below 0.05 were considered indicative of a significant difference between measurements.

## Results

### Transgenic Expression of Tyrosine Hydroxylase Is Mandatory for Dopamine Production in SH-SY5Y Cells

**Figure 1:**
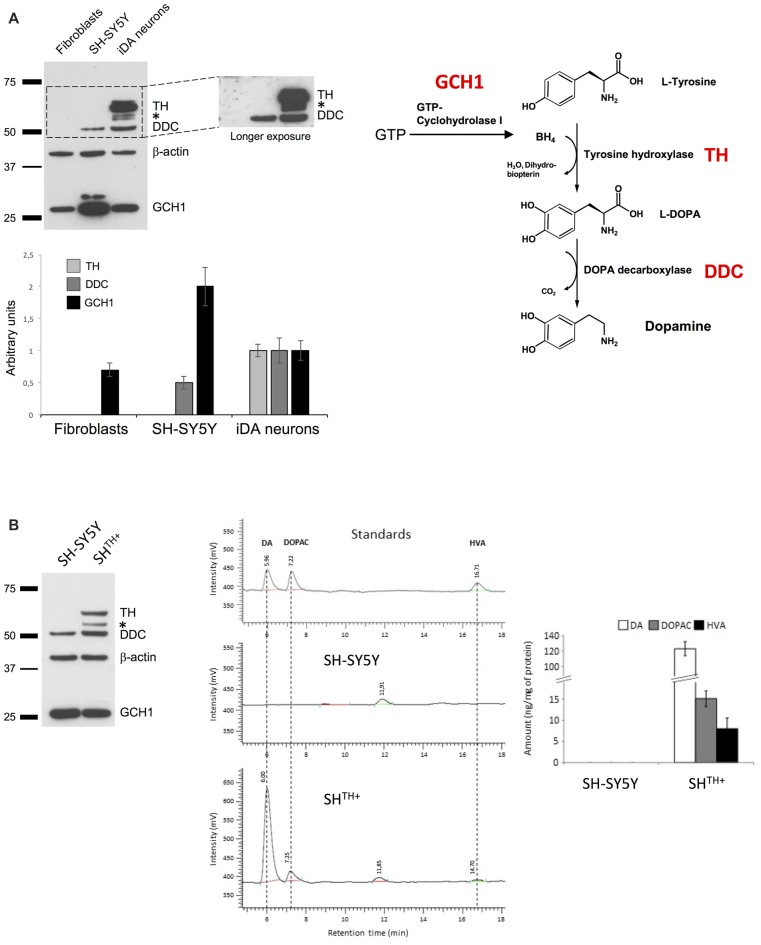
Ectopic expression of TH is sufficient for dopamine synthesis in SH-SY5Y cells. **(A)** Western blot analysis of DDC, TH and GCH1 in human dermal fibroblasts, SH-SY5Y cells, and induced pluripotent stem cells (iPSCs)-derived neurons. β-actin served as a loading control. **(B)** Proteins from naive SH-SY5Y and SH-SY5Y cells expressing TH (SH^TH+^) were extracted and analyzed using antibodies against TH, DDC and GCH1. Dopamine synthesis was detected only in SH^TH+^ cells, but not in SH-SY5Y cells. BH4, tetrahydrobiopterin; TH, tyrosine hydroxylase; L- DOPA, L-3,4-dihydroxyphenylalanine; DDC, DOPA decarboxylase; GCH1, GTP cyclohydrolase 1; *non-specific band.

While previous studies have shown that SH-SY5Y cells are able to produce DA, its levels and levels of its metabolites DOPAC and HVA were barely detectable. Therefore, we first sought to investigate the levels of the main enzymes involved in DA synthesis (Figure 1A), i.e., GCH1, TH and DDC in SH-SY5Y cells. As a positive control, we used previously differentiated iPSC-derived dopaminergic neurons (iDA neurons) originating from human dermal fibroblasts obtained from a healthy control (Rakovic et al., 2013). In addition, we used a human dermal fibroblast line that we employed to generate iDA neurons as a negative control (Figure 1A). As expected, in iDa neurons we detected all three enzymes, whereas human dermal fibroblasts expressed only GCH1. However, in SH-SY5Y cells we did not detect the presence of TH, even upon longer exposure, suggesting that TH is either not expressed at all or at very low levels only. To test whether transgenic expression of TH in SH-SY5Y cells would be sufficient to produce detectable levels of DA, DOPAC, and HVA, we generated SH-SY5Y cells stably expressing TH using selectable lentiviral particles generated using pLenti4-TH-IRES-Puromycin (SH^TH+^; Figure 1B). To quantify whether DA and its metabolites are present in SH^TH+^ cells, we conducted HPLC measurements for the detection of DA, DOPAC, and HVA. As indicated in Figure 1B, we were not able to detect any of these molecules in either non-transduced SH-SY5Y cells (SH-SY5Y) or in SH-SY5Y cells transduced with pLenti-Empty-IRES-Puromycin (data not shown). However, in SH^TH+^, we detected high levels of DA and of its metabolites suggesting that expression of TH was sufficient to reestablish dopaminergic metabolism in SH-SY5Y cells.

### Alteration of Monoaminoxidase Levels Increases DA Turnover in SH^**TH+**^ Cells

**Figure 2:**
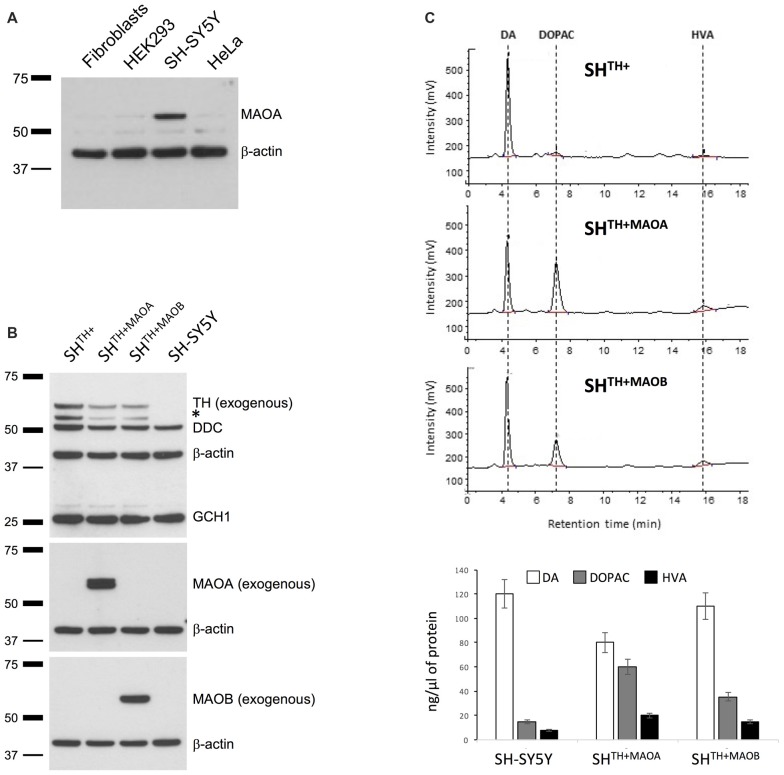
Monoamino oxidase A (MAOA) is expressed in SH-SY5Y cells. **(A)** Western blot analysis of MAOA in human dermal fibroblasts, SH-SY5Y cells, and iPSC-derived neurons. β-actin served as a loading control. **(B)** Western blot analysis of SH-SY5Y cells coexpressing TH and either MAOA (SH^TH+MAOA^) or MAOB (SH^TH+MAOA^). Naive SH-SY5Y and SH-SY5Y cells expressing TH only (SH^TH+^) served as controls. **(C)** High performance liquid chromatography (HPLC) analysis of DA, DOPAC, and HVA in SH^TH+^, SH^TH+MAOA^ and SH^TH+MAOB^. TH, tyrosine hydroxylase; DA, dopamine; DOPAC, 3,4-dihydroxyphenylacetic acid; HVA, Homovanillic acid; *non-specific band.

Normally, intracellular turnover of DA into DOPAC is regulated mainly through enzymatic activitiy of MAOA and only partially through activity of MAOB (Youdim and Bakhle, 2006). Therefore, we first confirmed the presence of endogenous MAOA by western blotting in SH-SY5Y cells but not in other non-neuronal, commonly used cell lines that served as negative controls (Figure 2A). This is consistent with our HPLC data showing the presence of DOPAC in SH^TH+^ under basal conditions (Figure 1B). We did not detect endogenous MAOB (data not shown), which is in keeping with previous findings showing that MAOA resides mainly in DA neurons and MAOB in microglia (Youdim and Bakhle, 2006). However, previous and recent studies showed a significant presence of MAOB in dopaminergic cells, as well (Damier et al., 1996; Woodard et al., 2014). To test whether our model indeed reacts to alterations in levels of MAOA and MAOB, we generated selectable SH^TH+^ cells stably overexpressing either MAOA (SH^TH+MAOA^) or MAOB (SH^TH+MAOB^) using lentiviral particles (Figure 2B). The levels of overexpressed MAOA and MAOB were confirmed by immunoblotting using antibodies specific for either MAOA or MAOB. Endogenous MAOA was not detected here due to high dilutions of the antibodies used to detect overexpressed proteins (Figure 2B). More importantly, in SH^TH+MAOA^ cells we detected a reduction in levels of DA and increased levels of DOPAC and HVA in comparison to non-transduced SH^TH+^ cells or SH-SY5Y cells transduced with lentiviral particles containing Empty-IRES-Blasticidin cassette (data not shown) indicative of MAOA-mediated conversion of DA into its metabolites. A similar, but much less pronounced degradation of DA was observed in SH^TH+MAOB^ cells (Figure 2C).

### CRISPR/Cas9-mediated Knock Out of DJ-1

**Figure 3:**
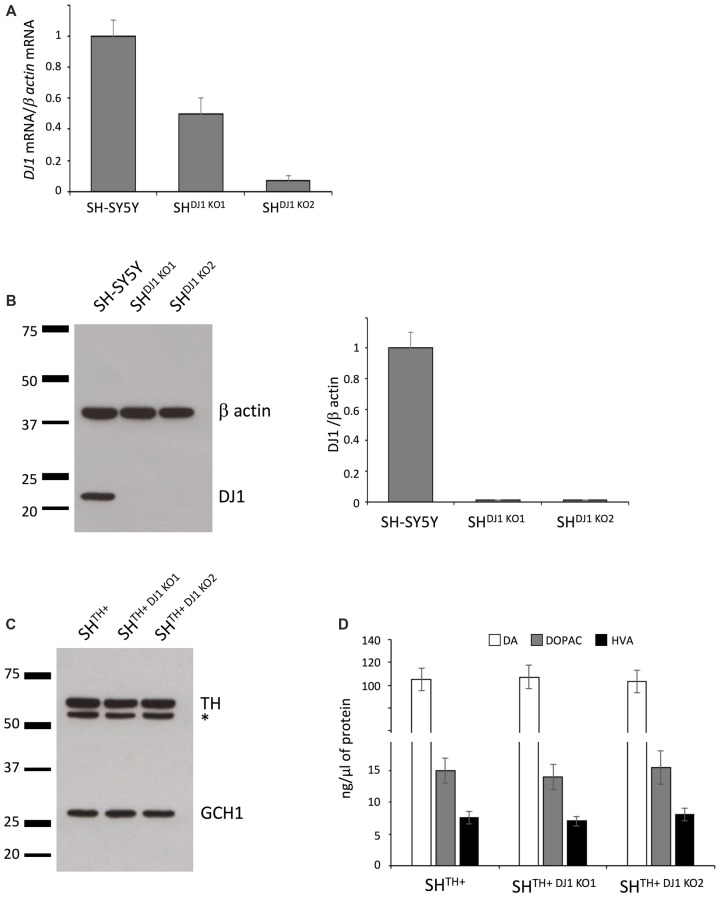
Knocking out DJ-1 has no influence on DA turnover. **(A)** *DJ-1* mRNA expression analysis in the wildtype (SH-SY5Y) and DJ-1 knockout SH-SY5Y cells (SH^DJ-1 KO1^ and SH^DJ-1 KO2^). **(B)** Western blot analysis of DJ-1 in SH-SY5Y, SH^DJ-1 KO1^ and SH^DJ-1 KO2^ showed complete loss of DJ-1 in both *DJ-1* knockout lines. β-actin served as a loading control. **(C)** Immunoblotting using antibodies against TH and GCH1 in SH^TH+^, SH^TH+DJ-1 KO1^ and SH^TH+DJ-1 KO2^ cells. **(D)** HPLC analysis showed no differences in levels of DA, DOPAC and HVA between wildtype and *DJ-1* knockout lines. TH, tyrosine hydroxylase; DA, dopamine; DOPAC, 3,4-dihydroxyphenylacetic acid; HVA, Homovanillic acid; *non-specific band.

We aimed at investigating whether loss of the recessively inherited PD-related gene DJ-1 would have an impact on DA turnover in our newly established model. To generate DJ-1 knockout SHSY5Y cells, we targeted the 5′-GTACAGTGTAGCCGTGATG-3′ sequence in exon 3 of the DJ-1 gene using CRISPR/Cas9 technology. Our sequencing data confirmed that we created two SH-SY5Y DJ-1 knockout lines, i.e., SH^DJ-1 KO1^ (a compound-heterozygous, c.135_146del12 + c.146delA) and SH^DJ-1 KO2^ (a compound-heterozygous, c.145_146insG, c.145_146insT). We observed a reduction in levels of DJ-1 mRNA in both lines, most likely caused by nonsense-mediated decay due to the out-of-frame indels in these lines. However, it appeared that the in-frame mutation c.135_146del12 had no effect on the levels of DJ-1 mRNA (Figure 3A). More importantly, when we analyzed protein levels of DJ-1 in both SH^TH+DJ1 KO1^ and SH^TH+DJ-1 KO2^, we detected a complete loss of DJ-1 protein in both knockout lines (Figure 3B).

Next, we transduced both DJ-1 knockout lines with lentiviral particles expressing TH to generate stable DA-producing SH-SY5Y DJ-1 knockout lines (SH^TH+DJ-1 KO1^ and SH^TH+DJ-1 KO2^). Comparable transgenic expression of TH was confirmed by immunoblotting (Figure 3C).

Finally, we analyzed levels of DA, DOPAC, and HVA in SH^TH+^, SH^TH+DJ-1 KO1^ and SH^TH+DJ-1 KO2^ cells using HPLC. Here, we did not observe any difference between wildtype and either of DJ-1 knockout lines in levels of either DA or its metabolites DOPAC or HVA (Figure 3D). Taken together, our data indicate that DJ-1 is not involved in DA synthesis or its turnover.

### Oxidative Stress in Dopaminergic Cells Lacking DJ-1

Catecholamine metabolism is a unique feature of catecholaminergic neurons and represents an additional source for ROS production (Meiser et al., 2013).

**Figure 4:**
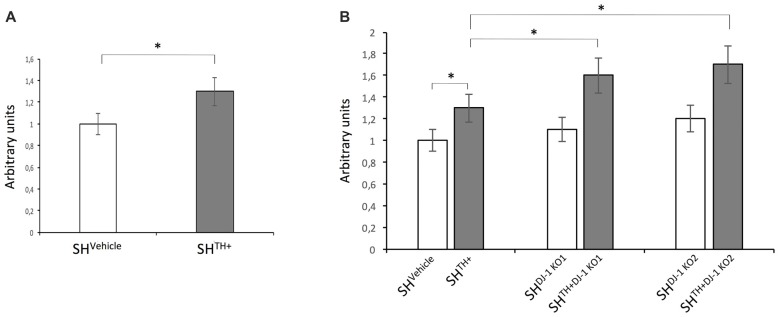
Loss of DJ-1 increases levels of oxidative stress in dopaminergic SH-SY5Y cells. **(A)** Levels of reactive oxygen species (ROS) in the SH-SY5Ycells stably expressing the empty-IRES-Puromycin cassette (SH^Vehicle^) and dopaminergic SH-SY5Y cells (SH^TH+^). **(B)** Levels of ROS were compared between DA-free SH^Vehicle^ and dopaminergic SH^TH+^ cells containing wildtype DJ-1 and DA-free (SH^DJ-1 KO1^ and SH^DJ-1 KO2^) and dopaminergic (SH^TH+DJ-1 KO1^, and SH^TH+DJ-1 KO2^) DJ-1 knockout cells. Levels of ROS in SH^Vehicle^ were set to 1. *p < 0.05.

To test whether the presence of DA and its metabolites increased oxidative stress in our dopaminergic cellular model, we compared levels of ROS between SH-SY5Y cells transduced with lentiviral particles expressing Empty-IRES-Puromycin cassette (SH^Vehicle^) and SH^TH+^ cells. We detected an increase in levels of ROS in SH^TH+^ in comparison to DA-free SH^Vehicle^ cells (Figure 4A). Previous studies in mice demonstrated a *DJ-1* knockout-related increase in levels of oxidative stress (Raman et al., 2013). Therefore, we aimed to investigate whether knocking out of *DJ-1* increased levels of ROS in both DA-free SH-SY5Y cells and dopaminergic SH^TH+^. In DA-free cells, we observed a slight but not significant increase in levels of ROS in both SH^DJ-1 KO1^ and SH^DJ-1 KO2^. On the other hand, loss of DJ-1 in dopaminergic cells, i.e., SH^TH+DJ-1KO1^ and SH^TH+DJ-1KO2^, induced a significant increase in levels of ROS in comparison to SH^TH+^ cells (Figure 4B). Please note that here we used SH^Vehicle^ for comparison to exclude a putative false-positive lentivirus-induced increase in levels of oxidative stress.

## Discussion

Although the definite disease mechanism underlying PD still remains a matter of extensive investigation, mitochondrial dysfunction, lysosomal and proteasome impairment, oxidative stress, or metal-ion dyshomeostasis have long been hypothesized to cause PD-specific degeneration of dopaminergic neurons (Dexter and Jenner, 2013). Nevertheless, most of these potential mechanisms failed to explain the specific vulnerability of dopaminergic neurons as the pathological hallmark of PD. Therefore, it is tempting to speculate that the aforementioned mechanisms contribute to the development of PD, but that there may be further cumulative effects connected to DA metabolism causing disease-specific neurodegeneration. Indeed, accumulation of oxidized dopamine results in reduced glucocerebrosidase enzymatic activity, lysosomal dysfunction, and α-synuclein accumulation (Burbulla et al., 2017) warranting further studies in a dopaminergic environment.

Recently, iPSC-based technology opened up new avenues to generate patient-derived DA neurons to study disease mechanisms in DA-producing cells but is expensive and technically challenging especially when it comes to the production of homogeneous neuronal cultures (Imaizumi and Okano, 2014). In addition, previous studies demonstrated that iPSC-derived DA neurons produce relatively low levels of DA not sufficient to effectively detect its subsequent metabolites, i.e., DOPAC and HVA.

In the present work, we used the SH-SY5Y neuroblastoma cell line as a well-established cellular model for neurological diseases. Using selectable lentiviral vectors, we were able to compensate for the lack of endogenous TH necessary for DA metabolism in SH-SY5Y cells and to create a homogenous DA-producing neuronal cellular system. Our model can be used to investigate existing or novel disease-causing mutations in TH, MAOs, and most likely other enzymes involved in DA metabolism by comparing cells expressing the wildtype and mutated forms of these enzymes using levels of DA as direct readout. We also demonstrated that our novel dopaminergic cellular system contains all components for DA synthesis and its metabolism. DA synthesis still remains within the physiological range; however, it was high enough to detect not only DA but also its subsequent metabolites, i.e., DOPAC and HVA.

The main limitation of our DA model is that, although neuronal, it does remain a tumor cell line. In contrast to neurons, the cells of our DA model are proliferative and express neuron-specific proteins at relatively low levels. However, these issues can be successfully overcome by (further) neuronal differentiation of neuroblastoma cells using retinoic acid (Kovalevich and Langford, 2013). Furthermore, we did not investigate whether our cellular model recapitulated important features of the dopaminergic system, i.e., whether DA is correctly packaged into DA vesicles and released upon a stimulus. In addition, we observed no changes in morphology of SH-SY5Y upon overexpression of TH. On the other hand, previous studies showed that even non-differentiated SH-SY5Y cells are suitable for experimental studies of neurodegenerative diseases in general, but more importantly, for PD in particular (Krishna et al., 2014).

Neuroblastoma cells can be relatively easily genetically modified to mimic human disease-causing mutations to investigate their role in a DA, neuronal cellular model. Here, as an example, we used the CRISPR/Cas9 system to examine the role of a recessively inherited PD-related gene, i.e., DJ-1 in DA synthesis and metabolism in our cellular model. We observed no influence of loss of DJ-1 on DA metabolism, or differences in levels of dopaminergic and its turnover in DJ-1 knockout cells in comparison to wildtype cells. However, we recapitulated the results of previous studies showing that loss of DJ-1 leads to increased levels of oxidative stress and that this increase becomes even more pronounced in a dopaminergic environment.

Taken together, we believe that our dopaminergic cellular model represents a significant improvement of an existing and commonly used cell line, i.e., SH-SY5Y cells, to investigate neurodegenerative disorders such as PD. In addition, our cellular system may serve as an affordable model for high-throughput studies to investigate DA metabolism and to identify potential therapeutic targets for subsequent evaluation in iPSC-derived neuronal or animal models.

## Author Contributions

JP, CK and AR: conception or design of the work and critical revision of the article. JP, CUM, VK and AR: data collection. JP and AR: data analysis, interpretation and drafting the article. JP, CUM, VK, CK and AR: final approval of the version to be published.

## Conflict of Interest Statement

The authors declare that the research was conducted in the absence of any commercial or financial relationships that could be construed as a potential conflict of interest.

## References

[B1] Burbulla L. F. Song P. Mazzulli J. R. Zampese E. Wong Y. C. Jeon S.. (2017). Dopamine oxidation mediates mitochondrial and lysosomal dysfunction in Parkinson’s disease. Science 357, 1255–1261. 10.1126/science.aam9080 28882997 PMC6021018

[B2] Damier P. Kastner A. Agid Y. Hirsch E. C. (1996). Does monoamine oxidase type B play a role in dopaminergic nerve cell death in Parkinson’s disease? Neurology 46, 1262–1269. 10.1212/WNL.46.5.1262 8628464

[B3] Dexter D. T. Jenner P. (2013). Parkinson disease: from pathology to molecular disease mechanisms. Free Radic. Biol. Med. 62, 132–144. 10.1016/j.freeradbiomed.2013.01.018 23380027

[B4] Imaizumi Y. Okano H. (2014). Modeling human neurological disorders with induced pluripotent stem cells. J. Neurochem. 129, 388–399. 10.1111/jnc.12625 24286589

[B5] Kovalevich J. Langford D. (2013). Considerations for the use of SH-SY5Y neuroblastoma cells in neurobiology. Methods Mol. Biol. 1078, 9–21. 10.1007/978-1-62703-640-5_2 23975817 PMC5127451

[B6] Krishna A. Biryukov M. Trefois C. Antony P. M. Hussong R. Lin J.. (2014). Systems genomics evaluation of the SH-SY5Y neuroblastoma cell line as a model for Parkinson’s disease. BMC Genomics 15:1154. 10.1186/1471-2164-15-1154 25528190 PMC4367834

[B7] Lill C. M. (2016). Genetics of Parkinson’s disease. Mol. Cell. Probes 30, 386–396. 10.1016/j.mcp.2016.11.001 27818248

[B8] Meiser J. Weindl D. Hiller K. (2013). Complexity of dopamine metabolism. Cell Commun. Signal. 11:34. 10.1186/1478-811X-11-34 23683503 PMC3693914

[B9] Postuma R. B. Berg D. Stern M. Poewe W. Olanow C. W. Oertel W.. (2015). MDS clinical diagnostic criteria for Parkinson’s disease. Mov. Disord. 30, 1591–1601. 10.1002/mds.26424 26474316

[B10] Rakovic A. Shurkewitsch K. Seibler P. Grünewald A. Zanon A. Hagenah J.. (2013). Phosphatase and tensin homolog (PTEN)-induced putative kinase 1 (PINK1)-dependent ubiquitination of endogenous Parkin attenuates mitophagy: study in human primary fibroblasts and induced pluripotent stem cell-derived neurons. J. Biol. Chem. 288, 2223–2237. 10.1074/jbc.M112.391680 23212910 PMC3554895

[B11] Raman A. V. Chou V. P. Atienza-Duyanen J. Di Monte D. A. Bellinger F. P. Manning-Bog A. B. (2013). Evidence of oxidative stress in young and aged DJ-1-deficient mice. FEBS Lett. 587, 1562–1570. 10.1016/j.febslet.2013.04.001 23587484

[B12] Woodard C. M. Campos B. A. Kuo S. H. Nirenberg M. J. Nestor M. W. Zimmer M.. (2014). iPSC-derived dopamine neurons reveal differences between monozygotic twins discordant for Parkinson’s disease. Cell Rep. 9, 1173–1182. 10.1016/j.celrep.2014.10.023 25456120 PMC4255586

[B13] Xicoy H. Wieringa B. Martens G. J. (2017). The SH-SY5Y cell line in Parkinson’s disease research: a systematic review. Mol. Neurodegener. 12:10. 10.1186/s13024-017-0149-0 28118852 PMC5259880

[B14] Youdim M. B. Bakhle Y. S. (2006). Monoamine oxidase: isoforms and inhibitors in Parkinson’s disease and depressive illness. Br. J. Pharmacol. 147, S287–S296. 10.1038/sj.bjp.0706464 16402116 PMC1760741

